# Electron conductive compounds alter fermentative pathways and cooperation in *Clostridium carboxidivorans* and *Clostridium acetobutylicum* in co-culture

**DOI:** 10.1093/femsec/fiaf090

**Published:** 2025-09-16

**Authors:** Laura Feliu-Paradeda, Sebastià Puig, Lluís Bañeras

**Affiliations:** Molecular Microbial Ecology Group, Institute of Aquatic Ecology, University of Girona, Carrer Maria Aurèlia Capmany 40, E-17003 Girona, Spain; LEQUiA, Institute of the Environment, University of Girona, Carrer Maria Aurèlia Capmany 69, E-17003 Girona, Spain; Molecular Microbial Ecology Group, Institute of Aquatic Ecology, University of Girona, Carrer Maria Aurèlia Capmany 40, E-17003 Girona, Spain

**Keywords:** *Clostridium*, co-culture, conductive materials, electron transfer, iron oxidation and reduction, magnetite

## Abstract

The addition of conductive materials promotes interactions between bacteria as they facilitate the exchange of reducing equivalents among cells. In this work, the impact of electron conductive compounds (magnetite, activated carbon, or iron salts) was investigated on a *Clostridium acetobutylicum/Clostridium carboxidivorans* co-culture. Co-culturing both species with soluble iron salts or magnetite significantly improved carbon recovery in liquid end-products (75%–85% of added carbon) compared to control and activated carbon supplementation (50%–55% of added carbon). The addition of magnetite enhanced the production of longer-chain acids and alcohols (C4 and C6) when compared to all other treatments and reached the highest production after 44 h of fermentation. This effect was not observed in *C. carboxidivorans* nor in *C. acetobutylicum* pure cultures, advocating for a cooperation between the two species. Among comparisons to the behaviour observed in pure cultures, we suggest magnetite was first used as a sink of reduced equivalents produced by *C. carboxidivorans* and later as a source of energy for *C. acetobutylicum* for the production of elongated short-chain fatty acids and alcohols. We propose that adding magnetite (iron) could be an effective strategy to enhance alcohol production in synthetic clostridia consortia.

## Teaser

The synergy between *C. acetobutylicum* and *C. carboxidivorans* is promoted in the presence of conductive materials, leading to higher CO2 recovery and production of longer carbon-chain alcohols.

## Introduction

*Clostridium* comprises multiple species, many of which can produce biofuels and other platform chemicals of great industrial interest. *Clostridium* metabolism and physiology, such as the acetone-butanol-ethanol (ABE) fermentation, has been widely studied for over a century. However, developing new strategies to improve fermentation yields is still necessary (Moon et al. 2016). Because of *Clostridium*’s flexible metabolic capabilities, one of the proposed strategies is co-culturing a solventogenic species with strains harbouring complementary metabolisms (Du et al. 2020). For example, combining a solventogenic strain, capable of producing solvents (i.e. acetone, butanol, and ethanol), with a cellulolytic strain, capable of hydrolysing complex polysaccharides into simple sugars, allows the production of butanol and other value-added compounds from lignocellulosic biomass. Additionally, the co-culture of a solventogenic strain with an acetogenic strain, which can grow autotrophically using the Wood-Ljungdahl pathway, could enhance carbon recovery and product yield, as the acetogen can capture the CO_2_ released during glycolysis (Cui et al. 2021).

Cooperation or metabolic dependency between cells should be necessary when aiming for stable consortia (Lovley 2016). In this sense, cell-to-cell interactions in consortia offer several synergistic benefits as cells coordinate their activity, growth, and movement. When co-cultured, cells can perform complementary functions by dividing the metabolic activity among them or match and/or enhance their growth by exchanging small metabolites or other small molecules and cytoplasmatic material, which is known as syntrophy or metabolic cross-feeding (Paquete et al. 2022). For example, in a syntrophic co-culture containing the solventogenic *C. acetobutylicum* and the acetogenic *C. ljungdahlii*, correlative fluorescence microscopy demonstrated cell fusion and exchange of proteins and RNA, which resulted in a superior metabolite yield and an optimal carbon and electron recovery (Charubin and Papoutsakis 2019, Charubin et al. 2020). Some microorganisms can even exchange electrons with either electron-donating or electron-accepting cells. This can occur via (i) soluble electron shuttles (e.g. formate, H_2_, or flavins), which enable extracellular electron transfer (EET) between cells without cell-to-cell contact; or (ii) with extracellular electrical interfaces (e.g. intracellular membranous nanotubes, pili, cytochromes) that facilitate cell-to-cell EET, which is known as direct interspecies electron transfer (DIET) (Lovley 2016, Rotaru et al. 2021, Paquete et al. 2022).

Abiotic conductive materials can be added to further promote electron transfer, a process known as conductive particle-mediated interspecies electron transfer, and can be performed by adding conductive carbon (e.g. granular activated carbon, biochar, or carbon cloth) or iron-based particles (e.g. magnetite, hematite, ferrihydrite) (Lovley 2016, Rotaru et al. 2021). Among conductive materials, magnetite (a mixed-valence iron oxide, Fe_3_O_4_) has been widely used to analyse electron shuttling events among microorganisms. Specifically, its addition has been observed to promote methanogenesis by facilitating electron transfer from a microbial partner species that oxidizes an organic substrate (Cruz Viggi et al. 2014, Paquete et al. 2022). Due to its high conductivity and iron abundance, magnetite supports electron transfer in a way comparable to the c-type cytochrome OmcS in *Geobacter sulfurreducens*, helping to minimize electron flow resistance (Jin et al. 2023). Although studies on the interaction between *Clostridium* sp. and magnetite are limited, most have focused on improving fermentative hydrogen production of pure strains. In those examples, the iron content of magnetite is suspected to support essential functions of critical hydrogen-producing enzymes (e.g. hydrogenases and ferredoxins) (Zhang et al. 2019, Kim et al. 2022, Mostafa et al. 2022, Mishra and Pradhan 2024). To the best of our knowledge, the impact of magnetite addition on *Clostridium* co-cultures, particularly in relation to metabolic synergies, remains unexplored.

In this study, the impact that the addition of electron-active compounds (semiconductor-like magnetite, weakly conductive activated carbon, and redox-active iron salts) has on the metabolic performance of a clostridial consortium containing the solventogenic *C. acetobutylicum* and the acetogenic *C. carboxidivorans* was evaluated. The aim was to investigate whether these conductive materials could act as electron shuttles, enhancing the interaction between species and thereby contributing to more efficient alcohol production in microbial fermentation. To achieve this, fermentation products obtained from *C. acetobutylicum/C. carboxidivorans* co-culture were compared with those obtained from each species in pure culture. Moreover, the extent of magnetite-positive action on alcohol production was also assessed by adding the cellulolytic *C. cellulovorans* in a triplet consortium.

## Materials and methods

### Bacterial strains, media, and cultivation conditions

*Clostridium carboxidivorans* P7 (DSM 15243), *Clostridium acetobutylicum* ATCC 824 (DSM 792), and *Clostridium cellulovorans* 743B *(*DSM 3052) were purchased from the Deutsche Sammlung von Mikroorganismen und Zellkulturen GmbH (DSMZ, Germany). All strains were activated using the recommended media and instructions (DSM 104c for *C. carboxidivorans*, DSM 104b for *C. acetobutylicum* and DSM 520 for *C. cellulovorans*). Stock cultures of the three strains were kept in anaerobic 25% glycerol and frozen at −80°C until fermentation experiments.

For fermentation experiments, pure cultures were pre-cultured in 50 ml of a defined consortia mineral medium (CM) containing (in 1 l): KH_2_PO_4_, 1 g; K_2_HPO_4_, 1 g; (NH_4_)_2_SO_4_, 1 g; FeSO_4_ × 7H_2_O, 0.001 g; MgCl_2_ × 6H_2_O, 0.2 g; CaCl_2_ × 2H_2_O, 0.075 g; trace element solution SL-10, 1 ml; Na-resazurin, 0.5 mg; yeast extract, 1.5 g; L-cysteine × HCl, 0.45 g; Na_2_CO_3_, 1.5 g. Glucose was supplemented in the CM at an approximate concentration of 5 g l^−1^ (which ranged from 4.1–4.6 g l^−1^ in *C. acetobutylicum*, 4.5–6.3 g l^−1^ in *C. carboxidivorans*, and 4.1–4.6 g l^−1^ in co-culture serum bottles) and used as the carbon source. For carbon and electron balances, the actual glucose concentration added to each bottle and condition was used (see below). Cysteine, sodium carbonate and glucose were added from anoxic filtered stock solutions after sterilization of the basal medium.

Pure strains were incubated overnight (approximately for 12 h) at 37°C. Optical densities were measured to estimate the cell density, and a mixture of *C. acetobutylicum* and *C. carboxidivorans* was prepared, aiming at having a 1:1 ratio of each strain in the inoculum at the beginning of the fermentation experiment. After that, a 10% (v: v) inoculation was performed in freshly prepared media. Bottles were flushed with a CO_2_: H_2_ (20:80) gas mixture for 5 min and kept at an initial pressure of 1.4 Bar (total pressure). Cultures were incubated at 37°C ± 1°C at a constant agitation of 130 rpm in a Stuart SI500 incubator (Bibby Scientific Limited, OSA, UK) to facilitate gas transfer between headspace and culture. All fermentation experiments were run in triplicate in serum bottles. The same procedure was used for the triplet consortium containing *C. acetobutylicum, C. carboxidivorans*, and *C. cellulovorans*, except that 5 g l^−1^ of cellobiose were used as the carbon source instead of glucose.

### Magnetite, activated carbon, and iron addition

Batch experiments were conducted in 250 ml anaerobic serum bottles sealed with butyl rubber septa and capsulated with aluminium crimps, with a final working volume of 100 ml of CM. Magnetite was supplemented to serum bottles (MAG treatment) from a sterile anaerobic magnetite solution at a final concentration of 0.42 g Fe_3_O_4_ l^−1^ (corresponding to a concentration of 0.3 g Fe l^−1^), as performed in Cruz Viggi et al. (2014). Magnetite solution was synthesized in the laboratory following the protocol from Kang et al. (1996). Before supplementation, the magnetic properties of the synthesized magnetite were confirmed. Granular activated carbon (CalgonCarbon https://www.calgoncarbon.com/, mean diameter 0.3 mm) was added at a final concentration of 0.84 g l^−1^ (CA treatment) to ensure a higher surface to volume ratio due to the larger particle size. The mixture of dissolved iron chloride (FeCl_2_/FeCl_3_, 50/50 w/w) was added at a final concentration of 0.3 g Fe l^−1^ (FE treatment). A control (C) treatment without any supplementation was included. Both carbon and iron chloride were added directly to the medium before autoclaving—even though the addition of iron salts before autoclaving is risky, as it can precipitate as various poorly soluble salts, or even catalyze the formation of some magnetite potentially creating conditions slightly similar in FE to those in MAG treatment.

### Analytical methods

Gas pressure and composition were analyzed at the beginning and at the end of the experiments. Gas pressure was measured using a digital pressure sensor (differential pressure gauge, Testo 512, Spain). Gas composition was analyzed by gas chromatography using a 490 Micro GC system (Agilent Technologies, USA) coupled to a thermal conductivity detector (TCD) and equipped with two columns: a CP-molesive 5A for CO, H_2_ and N_2_; and a CP-Poraplot U for CO_2_ analysis.

Liquid samples were taken periodically to measure pH, cell density and fermentation products. Samples were centrifuged at 12 000 rpm for 2 mins. Pellets were kept frozen at −20°C for DNA extraction. Supernatants were filtered through a 0.22-µm pore-size filter and used to analyze the concentration of alcohols (i.e. ethanol, butanol, and hexanol), volatile fatty acids (i.e. acetic acid, butyric acid, and caproic acid), and sugars (i.e. cellobiose and glucose). Sugars were analyzed by an ionic chromatograph (Dionex ICS5000, USA) equipped with a Carbopac PA20 column and an electrochemical detector. Acids and alcohols were measured by gas chromatography using an Agilent 7890A (Agilent Technologies, USA) equipped with a DB-FFAP column and coupled to a flame ionization detector.

### Calculations and statistics

The percentage of electrons recovered into dissolved products (i.e. alcohols and volatile fatty acids) was calculated according to input sources (i.e. glucose, H_2_, and Fe^2+^) and the increase in product concentration at the end of the incubation. Similarly, for C-balance estimation, only glucose, carbonate, and CO_2_ were considered as carbon sources. Estimations were calculated using the initial and end concentrations and the electron equivalents assignments as reported by Li et al. (2024b). Glucose, cellobiose, H_2_ and Fe^2+^ contributed 24, 48, 2, and 1 mol electron equivalents (e^-^) per mol of the compound, respectively. Electron equivalents recovered in products were considered to be 12 e^-^, 24 e^-^, 36 e^-^, 8 e^-^, 20 e^-^, and 32 e^-^ for ethanol, butanol, hexanol, acetate, butyrate, and hexanoate, respectively. SPSS software (IBM) was used to test for statistically significant differences in alcohol and acid production between experimental conditions within each consortium or monoculture at 22 h or 44 h. Comparisons were performed independently within the same time point (22 and 44 h) and per compound (acetate, butyrate, caproate, and ethanol, butanol, and hexanol). When comparing results across multiple conditions (C, MAG, AC, FE), an ANOVA (with Bonferroni correction) or a Kruskal–Wallis test (if homoscedasticity could not be assured) was performed. For comparisons in the triplet consortium between control and magnetite, either a *t*-test (for normally distributed data with equal variances) or a Mann–Whitney U test (for non-normally distributed data) was applied. Dunn’s test was applied for carbon and electron balance comparisons.

### Quantification of cell density by qPCR

DNA was extracted from cell pellets using Chellex^®^ resin using the manufacturer’s recommended method for bacteria. Cell density of the three species, separately, was quantified by using a multiplex quantitative polymerase chain reaction (qPCR) assay as previously described (Feliu-Paradeda et al. 2023). Briefly, multiplex qPCR assays were performed in a LightCycler 96 Real-Time PCR instrument, using the primer pair ClosF (5′-CGAAAGGGAGATTAATACCGCA-3′) and ClosR (5′-GAGCCGTTACCTCACCAAC-3′), and species-specific TaqMan probes – Clocar (6FAM-TAAAGGAGTAATCCGCTTTGAGATGGGC-BHQ1), Cloace (Cy5-TGATTCTTGAGCCAAAGGATTTATTCGC-BHQ2), and Clocel (VIC-CGCATGAGAGATGTATCAAAGGAGCAAT-BHQ1) –, in a 20 µl reaction containing: 1 µl of DNA template, 10 µl of FastStart Essential DNA Probes Master 2X (Roche Diagnostics, Germany), 6 µl of DEPC water, and 3 µl of Assay Mix containing the corresponding TaqMan probe and primer pair (with a final reaction concentration of 0.25 µM of probe and 0.90 µM of each primer). Conditions were the following: a preincubation step at 95°C for 5 mins, followed by 45 cycles of a two-step amplification (denaturalization at 95°C for 10 s, annealing and extension at 61°C for 30 s). Each qPCR reaction was performed in triplicates. Cell concentrations were calculated from 16S rRNA gene copies considering the number of copies present in the genome of the three species (9 copies in *C. cellulovorans*, and 11 copies in *C. acetobutylicum* and *C. carboxidivorans*).

### Scanning electron microscopy analysis

Scanning electron microscopy (SEM) imaging was used to check for the formation of cell aggregates and cell adhesion on magnetite or activated carbon particles. After fermentation experiments, particles were allowed to sediment to the bottom of bottles. Afterwards, 0.5 ml samples were collected, dispensed in Eppendorf tubes and centrifuged for 10 min at 4°C at 2.200 rpm. After removing the supernatant, the remaining pellet was resuspended with 1 ml of glutaraldehyde (2.5% w/v) and incubated at 4°C for 4 h, centrifuged, and washed twice with 1 ml of cacodylate 0.1 M. After cacodylate, samples were washed with water and dehydrated in an ethanol series of 50, 75, 90, and 4 times 100% steps (20 min each) and dried with a CO_2_ critical point dryer (model K-850 CPD, Emitech, Germany). Dried samples were stabilized with a carbon bio-adhesive and sputtered-coated with a 40 nm gold layer. The coated samples were examined with SEM (AXS Micro-analysis GmbH, Germany) at 15 keV. The analysis was performed in the facilities of the Serveis Tècnics de Recerca, University of Girona.

## Results

### Effects of magnetite, activated carbon, and iron salts on mono-culture and co-culture fermentation

**Figure 1.**
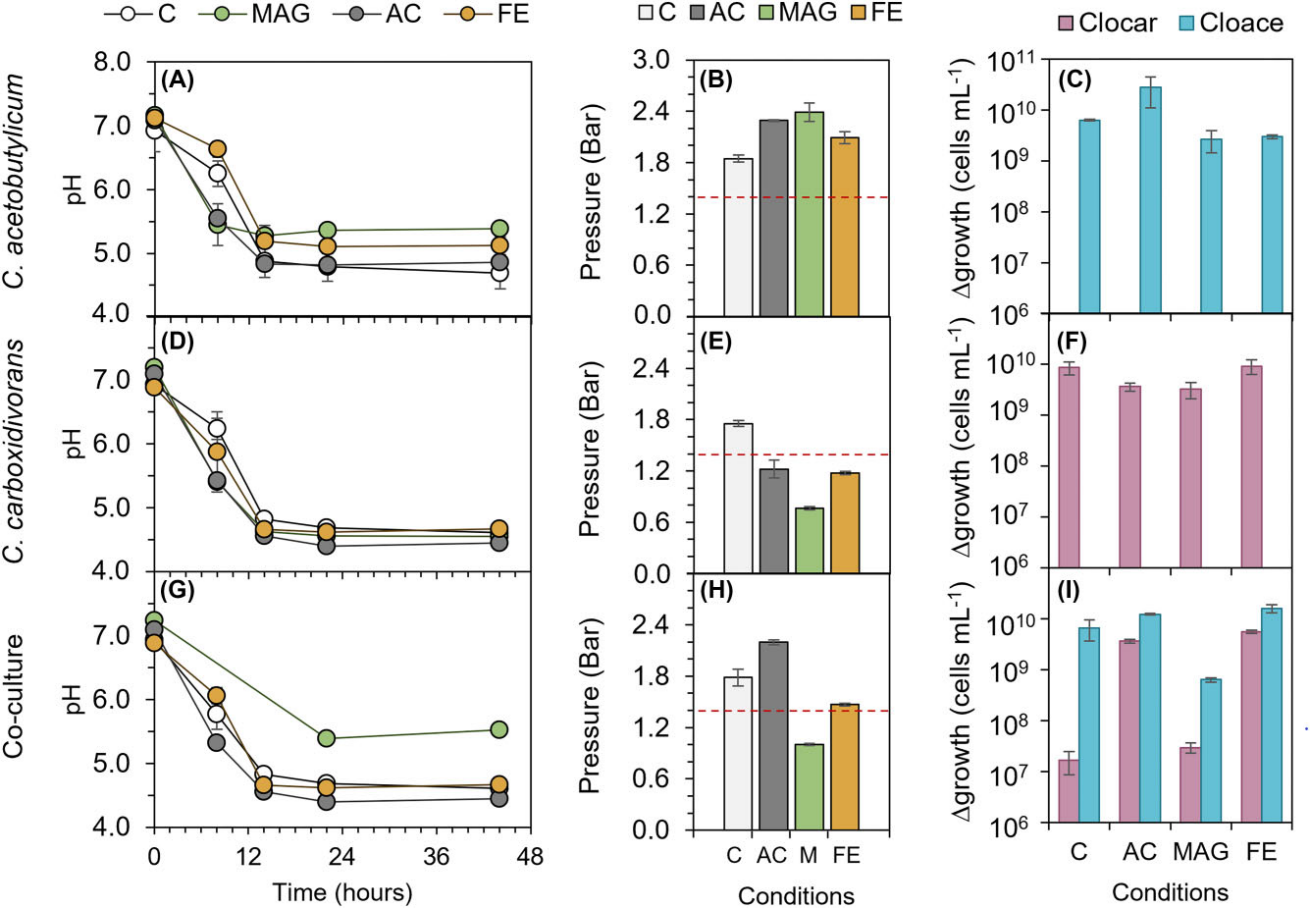
pH evolution, gas pressure and increment of *Clostridium* cell numbers in *C. acetobutylicum* (A, B, C), *C. carboxidivorans* (D, E, F), and their co-culture (G, H, I). Abbreviations are as follows: C, control (without materials); MAG, magnetite; AC, activated carbon; FE, iron chloride; Cloace, *C. acetobutylicum*; Clocar, *C. carboxidivorans*. Dashed line in gas pressure graphics (B, E, and H) indicate the gas pressure at the beginning of fermentation.

Fermentation performance in terms of growth and product formation was analysed in pure cultures of *C. acetobutylicum* and *C. carboxidivorans* and in *C. acetobutylicum/C. carboxidivorans* co-culture in the presence of magnetite, activated carbon and iron salts (Fig. 1). Glucose, used as the carbon source, was fully consumed within 22 h after inoculation, independently of the culture and the supplemented electron active compound. In *C. acetobutylicum* culture, pH values dropped below 4.8 in control and AC treatment but remained above 5.2 in MAG treatment (Fig. 1A). Gas production and cell growth were detected in all four conditions (Fig. 1B, C). Pure cultures of *C. carboxidivorans* reached similar pH values at the end of fermentation independently of the treatment applied, even though the pH dropped slightly faster in MAG and AC treatments (Fig. 1D). Contrarily to what was observed for *C. acetobutylicum*, overpressure was only detected in the control treatment, indicating that all supplementations enhanced autotrophic metabolism in this strain (Fig. 1E). The most pronounced differences were observed in the magnetite-supplemented treatment. Nonetheless, the extra captured CO_2_ was not translated into a higher cell density since no significant differences in cell densities were found among treatments (Fig. 1F).

In the co-culture, the growth behaviour resulted in a combination of the results observed for the separate species. AC treatments behaved similar to *C. acetobutylicum* experiments, i.e. high overpressure at the end of the fermentation period and rapid pH drop. In contrast, MAG and FE treatments showed an increased consumption of gas compared to the control experiment, thus suggesting the onset of autotrophic carbon fixation by *C. carboxidivorans* (Fig. 1H). Despite the lower pressure of the headspace in MAG bottles, the gas was enriched in CO_2_, which increased from 20% to 30% by the end of the experiment. At that point, 3.8 mmol H_2_ were found in the gas phase of MAG bottles, lower compared to all the other treatments (7.8, 9.3, and 6.2 mmol H_2_ in control, AC, and FE, respectively), indicating magnetite promoted the consumption of H_2_. Despite the similarities with the results obtained for *C. carboxidivorans* in pure culture, the pH value of the co-culture remained at 5.5 in the MAG treatment, well above the average value for the other treatments (Fig. 1G). Stationary phase CO_2_ consumption in MAG treatments with the co-culture was likely directed to the formation of products rather than cell growth since cell numbers remained at significantly lower values compared to FE treatment (Fig. 1I).

**Figure 2.**
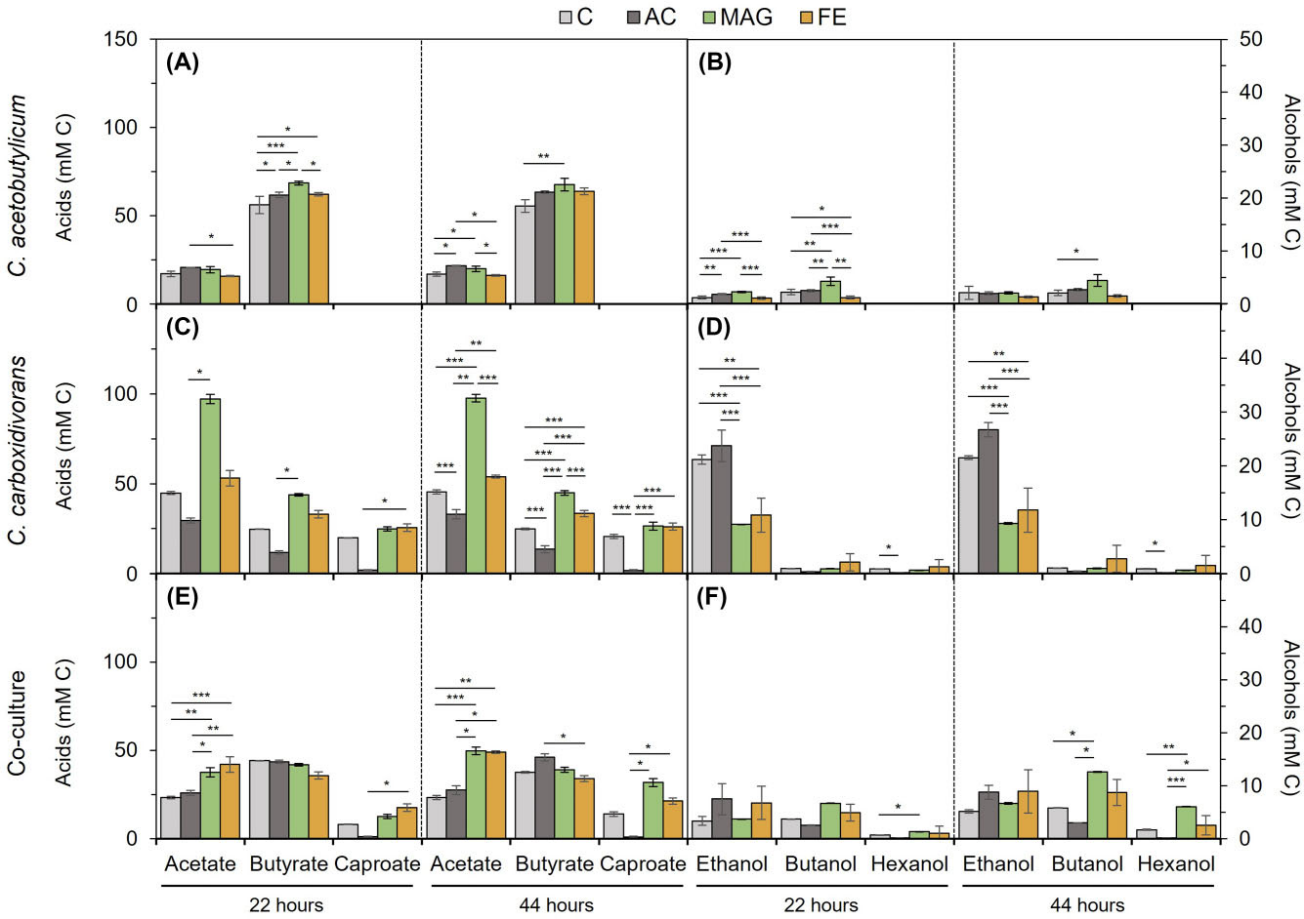
Concentration of acids (left) and alcohols (right) produced by *C. acetobutylicum* (A, B), *C. carboxidivorans* (C, D), and their co-culture (E, F) after 22 and 44 h of fermentation experiments. Abbreviations are as follows: C, control; AC, activated carbon; MAG, magnetite; FE, iron chloride. Mean values and standard errors of three replicates are shown in all graphs. Statistically significant differences between conditions within the same sample point (according to Bonferroni or Kruskal–Wallis) are indicated with asterisks, with *P-value ≤ 0.05, **P-value ≤ 0.01, and ***P-value ≤ 0.001. Note *Y*-axis differs in acids and alcohols.

The production profiles of pure cultures and the co-culture differed according to the applied treatments, although the changes were more pronounced in the co-culture (Fig. 2). In *C. acetobutylicum* pure cultures, FE, CA, and MAG addition caused a small effect on the product spectrum and product yield. Acetate and butyrate concentrations appeared to be higher in AC and MAG treatments, whereas butanol was significantly higher (216%) in MAG compared to control (4.39 and 2.06 mM C, respectively) but not in AC and FE treatments (Fig. 2A, B). Collectively, the used treatments showed little effect on *C. acetobutylicum* metabolism. The product spectrum and final concentration in *C. carboxidivorans* pure cultures exhibited significant differences according to the applied treatment (Fig. 2C, D). Notably, C6 products (caproate and hexanol) were almost absent in the AC treatments (<4% C in products) despite showing a tendency to increase towards the 44 h of incubation. Alcohol production (mainly ethanol) decreased significantly by a factor of two, whereas acetate increased 2-fold in MAG and FE treatments. Maximum concentrations of ethanol were 9.31 ± 0.16 and 11.78 ± 4.12 mM C in MAG and FE treatments, compared to 21.52 ± 0.33 and 26.70 ± 1.30 mM C in control and AC treatments. The lowest acetate concentration was obtained in AC treatment (33.08 ± 2.49 mM C) but was significantly higher in control and FE treatments (45.53 ± 1.16 and 53.92 ± 0.81 mM C, respectively), and specially in MAG treatment (97.71 ± 2.07 mM C).

In *C. carboxidivorans/C. acetobutylicum* co-culture (Fig. 2E, F), butanol and hexanol production in MAG treatment significantly improved up to 214 and 351%, respectively, compared to control, as butanol and hexanol concentrations in MAG bottles after 44 h reached 12.04 ± 1.35 and 5.60 ± 1.75 mM C, respectively. Besides, a significant increase of acetate (from 20.43 ± 2.64 to 48.38 ± 10.32 mM C, 237% increase) was also observed, while butyrate levels showed no significant difference compared to the control. The addition of AC did not improve the production of acid or alcohols compared to control fermentation. Notably, the total production of acids and alcohols was similar in both conditions (AC and control), but the relative concentrations of the products differed. Supplementation of AC slightly improved the production of ethanol (8.74 ± 0.41 mM C) compared to control (5.14 ± 1.64 mM C) but reduced the production of caproate and hexanol (0.91 ± 0.18 and 0.14 ± 0.02 mM C, respectively, <2% in C products). FE and MAG treatments resulted in similar behaviour of the co-culture. A significant increase in the production of acetate (210%), caproate (153%) and hexanol (150%) was found in the FE treatment compared to control. In contrast, despite increasing during fermentation, differences in the butanol concentrations were not significantly different. Interestingly, alcohol production in the co-culture was sustained during the whole growing period (44 h) in MAG and FE contrasting AC treatment in which production was strictly related to cell growth. Collectively, the use of the co-culture in the presence of magnetite increased the concentration of butanol and hexanol, compared to *C. carboxidivorans* and *C. acetobutylicum* pure cultures (Table 1).

**Table 1.** Carbon and electron equivalents recovered in acids and alcohols in *C. acetobutylicum* and *C. carboxidivorans* pure cultures, the co-culture, and the triplet consortium.

		Control		AC		MAG		FE	
Culture	Products	Carbon (mmols C)^a^	Red. eq. (mols e^−^)^b^	Carbon (mmols C)^a^	Red. eq. (mols e^−^)^b^	Carbon (mmols C)^a^	Red. eq. (mols e^−^)^b^	Carbon (mmols C)^a^	Red. eq. (mols e^−^)^b^
*C. acetobutylicum*	Acids	6.82 ± 0.49 (42%)	32.70 ± 2.32 (44%)	8.11 ± 0.07 (50%)	38.62 ± 0.41 (51%)	8.37 ± 0.59 (54%)	40.11 ± 2.78 (55%)	7.38 ± 0.45 (49%)	36.95 ± 0.19 (53%)
	Alcohols	0.41 ± 0.07 (3%)	2.47 ± 0.42 (3%)	0.33 ± 0.06 (2%)	2.01 ± 0.34 (3%)	0.52 ± 0.15 (4%)	3.13 ± 0.88 (5%)	0.23 ± 0.05 (1%)	1.52 ± 0.32 (2%)
*C. carboxidivorans*	Acids	8.70 ± 0.20 (37%)	39.91 ± 1.09 (39%)	4.39 ± 0.45 (28%)	19.01 ± 2.04 (28%)	16.46 ± 0.37 (76%)	73.73 ± 1.94 (75%)	10.77 ± 0.72 (65%)	51.96 ± 0.74 (69%)
	Alcohols	2.15 ± 0.07 (9%)	12.93 ± 0.39 (13%)	2.59 ± 0.11 (16%)	15.56 ± 0.63 (21%)	0.95 ± 0.10 (4%)	4.35 ± 2.94 (6%)	1.42 ± 1.20 (9%)	5.47 ± 0.53 (7%)
Co-culture	Acids	6.78 ± 0.05 (43%)	32.32 ± 0.51 (45%)	7.05 ± 0.33 (48%)	32.79 ± 1.52 (49%)	11.44 ± 1.91 (71%)	53.57 ± 8.30 (71%)	9.67 ± 0.54 (62%)	43.75 ± 0.32 (61%)
	Alcohols	0.99 ± 0.48 (6%)	5.96 ± 2.91 (8%)	1.05 ± 0.77 (7%)	6.30 ± 0.42 (9%)	2.31 ± 0.32 (14%)	13.89 ± 1.90 (18%)	1.83 ± 1.04 (13%)	7.96 ± 0.54 (11%)
Triplet	Acids	8.22 ± 0.20 (50%)	38.91 ± 1.12 (52%)	-	-	12.69 ± 0.15 (78%)	59.15 ± 0.94 (80%)	-	-
	Alcohols	0.39 ± 0.19 (2%)	2.34 ± 1.09 (3%)	-	-	1.67 ± 0.05 (10%)	10.04 ± 0.28 (14%)	-	-

a The amount of carbon recovered in acids (acetate, butyrate, caproate) or alcohols (ethanol, butanol, hexanol) in penicillin bottles, containing 100 ml of medium. The percentage denotes the carbon recovered from carbon source and was calculated as the mmols of C in acids or alcohols divided by the mmols of C from the source, multiplied by 100.

b The amount of electron equivalent recovered in acids (acetate, butyrate, caproate) or alcohols (ethanol, butanol, and hexanol) in penicillin bottles containing 100 ml. The percentage value denotes the portion of the electron equivalent recovered from the electron input (from glucose and H_2_, plus Fe^2+^ in MAG and FE). AC (activated carbon), MAG (magnetite) and FE (iron chloride).

### Integration of *Clostridium cellulovorans* into the co-culture of *C. acetobutylicum/C. carboxidivorans* with magnetite

**Figure 3.**
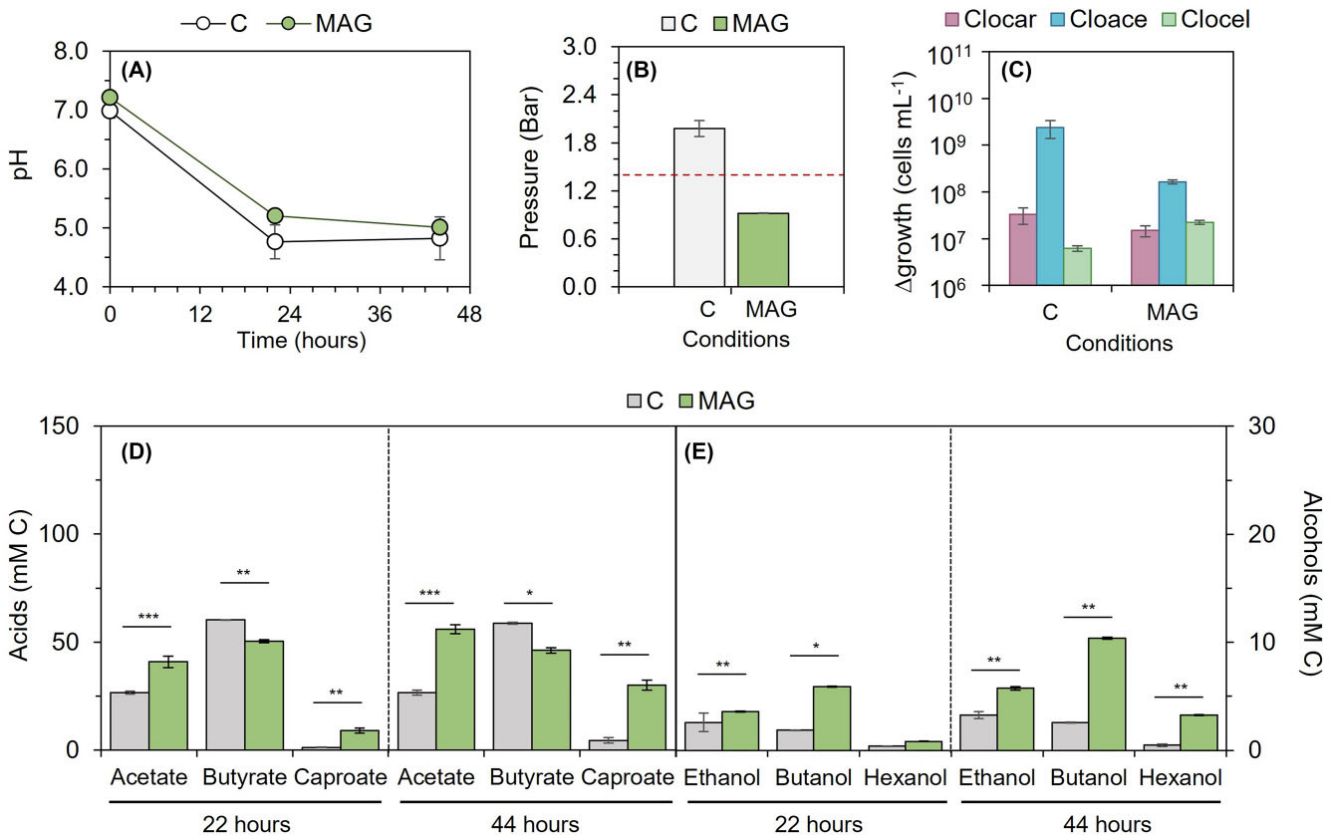
pH evolution (A), gas pressure (B), increment of *Clostridium* cell numbers (C), concentration of acids (D) and alcohols (E) produced by the triplet consortium. Bars show mean value of 3 replicates, and error bars indicate SD. Statistically significant differences (according to *t*-test or Mann–Whitney U test) are indicated with asterisks, with *P-value ≤ 0.05, **P-value ≤ 0.01, and ***P-value ≤ 0.001. Abbreviations are as follows: C, control; MAG, magnetite; Clocar, *C. carboxidivorans*; Cloace, *C. acetobutylicum*; Clocel, *C. cellulovorans*. Note *Y*-axis differs in acids and alcohols. Note *Y*-axis differs in acids and alcohols.

The positive influence of magnetite supplementation on alcohol production was also investigated in a triplet consortium containing *C. acetobutylicum, C. carboxidivorans*, and the cellulolytic *C. cellulovorans* (Fig. 3). The aim of this triplet consortium was to include a bacterium which potentially uses cellulosic materials. Cellobiose, which can be used as carbon and energy sources by the three co-cultivated strains, was used as the carbon source. Results obtained with the triplet consortium were similar to those obtained with *C. acetobutylicum/C. carboxidivorans* co-culture. The headspace of magnetite-supplemented bottles was slightly below atmospheric pressure at the end of the fermentation (Fig. 3B), suggesting *C. carboxidivorans* effectively fixed the CO_2_ and H_2_ produced during sugar fermentation. Again, despite the lower pressure of the headspace, the gas was enriched in CO_2_ (from 20% to 31% by the end of the experiment), which was likely directed to products rather than cell growth, as *C. carboxidivorans* cell numbers remained at lower values compared to control (Fig. 3C). Regarding product formation, the triplet consortium showed significant improvements in the production of ethanol (175% increase), butanol (407%), hexanol (690%), acetate (209%), and caproate (667%), as higher concentrations were produced in the presence of magnetite compared to control (Fig. 3D, E). The production of acids and alcohols was sustained in MAG treatments, contrarily to control bottles where the production remained stable after 22 h of incubation, thus revealing that the effect of magnetite was prolonged after the complete depletion of sugar that occurred concomitantly with the exponential phase (before 20 h after inoculation). Altogether, introducing *C. cellulovorans* did not influence the interaction between *C. acetobutylicum* and *C. carboxidivorans* in the presence of magnetite.

### Carbon and electron balances

Carbon and electron balances were used to investigate the changes in the distribution of reducing equivalents and the potential disruptions caused by the addition of conductive compounds. Carbon recovery in control experiments was about 45% in pure cultures and slightly increased to 49% in the co-culture, and to a 52% in the triplet consortium (Table 1). Similar variations were found for the electron equivalents distribution, indicating co-culturing resulted in limited improvements in carbon transformation into products. In *C. acetobutylicum*, the carbon and electron conversion to liquid end-products remained relatively stable across all treatments, with 45 to 58% of the carbon directed to mainly acids and no significant differences were found among treatments (*P* > 0.05 Dunn test). In *C. carboxidivorans*, changes in the carbon and electron distribution were found according to treatments indicating an active role of conductive materials in the fermentation behaviour of this organism. Significantly higher amount of carbon was directed to product formation, that increased in MAG (80% of added carbon) and FE (74%) treatments, respectively. The changes were accompanied by a significant reduction of the recovered reducing equivalents in alcohols, changing from 13% to 21% in control and AC treatments to less than 7% in MAG and FE, indicating iron containing compounds acted as an electron sink. As shown previously, the concentrations of butyrate and caproate increased significantly in those treatments. Significant increases on the amount of carbon and electrons fixed into soluble products in comparison to control were also found in the co-culture when MAG and FE were added (*P* < 0.01 Dunn test). Notably, the production of alcohols was higher, and the electron equivalents fixed increased from 8% (control) to 18% (MAG) or 11% (FE). An equivalent effect was observed in the triplet consortium in which reducing equivalents recovered increased from 55% (17:1 acid to alcohol ratio) to almost 94% (6:1 acid to alcohol ratio) indicating the presence of a cellulose-degrading bacterium in the consortium was not affecting cooperation negatively.

## Discussion

Co-cultivation of a model ABE fermenter and an autotrophic acetogenic clostridium was tested as a means to recover the wasted CO_2_ and increase metabolic carbon yield into products during sugar fermentation. The co-metabolic interaction between the two species was modulated after adding particulate or soluble electron-active compounds, such as activated carbon, magnetite or dissolved iron. As shown previously, the co-culture exhibited changes in the product spectrum and concentration when compared to the individual species. Co-culturing of *C. carboxidivorans* and *C. acetobutylicum* in the presence of Fe containing compounds (MAG and FE treatments) caused a shift to the production of alcohols that was not observed in the pure cultures. Specifically, acids to alcohols ratio was 17.3:1 and 7.6:1 (mmol C:mmol C ratio) in MAG and FE respectively when *C. carboxidivorans* was cultured alone, and shifted to 5.0:1 and 5.3:1 mmol C:mmol C ratio in MAG and FE, respectively, when co-cultured with *C. acetobutylicum* showing a significant enrichment in alcohol production. This effect suggests a metabolic interaction between the two partners mediated by the presence of an external electron donor/acceptor since changes in the control treatments were not observed.

To serve as electron conduits and facilitate electron transfer, conductive materials and cells need to be in contact (Kato et al. 2012, Rotaru et al. 2014, Lovley 2016). Scanning electron microscopy images of AC and MAG particles after fermentation experiments revealed that cells were tightly associated with the surface of the two materials (Supplementary Fig. S1). A similar behaviour has been observed in other studies (Liu et al. 2012, Cruz Viggi et al. 2014). In Liu’s and coworkers’ contribution, metabolic stimulation of the consortium partners is discussed in terms of DIET events, in which activated carbon may provide a higher conductivity between cells, thus avoiding the need for biologically produced electrical connections (Liu et al. 2012). In our work, co-culturing and the addition of AC to the consortium did not yield any significant effect in terms of production, thus suggesting direct electron transfers between cells could be discarded for the two used clostridia. Cruz-Viggi and coworkers demonstrated that magnetite supplementation could reinforce electrical connections between bacterial-archaeal partners increasing overall methane production by a 30% and supporting the view of better optimization of reducing power equivalents in the two partners (Cruz Viggi et al. 2014). The above examples explore DIET mechanism as a plausible mechanism and use confirmed electrogenic bacteria (i.e. *Geobacter* spp.) as a key member of the consortium. The capacity of *Geobacter sulfurreducens* to transfer electrons derived from the oxidation of acetate to a graphite electrode was confirmed >20 years ago (Bond and Lovley 2003), and more recently the molecular mechanism for the transfer has been proposed involving outer membrane multi-heme cytochromes (MHC) that can be structured into filaments (Butler et al. 2010, Salgueiro et al. 2022). In fact, the presence of MHC has been confirmed in *C. aceticum* but not in other clostridia, such as *C. pasteurianum, C. acetobutylicum*, and *C. ljungdahlii*, despite they have been described as potentially electrotrophic (Logan et al. 2019, Garber et al. 2024). Nevertheless, this remains as a hypothesis since at least for *C. ljungdahlii* a confirmation for its dependence on H_2_ and not on direct electron capture when grown in bioelectrochemical systems has been reported (Boto et al. 2023). Production capacities in the co-culture amended with AC did not differ significantly compared to the control conditions, and the inclusion of redox-active iron-containing molecules (MAG and FE treatments) was necessary, thus limiting the extent of true electron translocating events between the two species tested here. This could be observed by the fact that a clear change in the colour of the medium could be observed after inoculation of the co-culture in the presence of magnetite (Supplementary Fig. S2). While a dark grey colour was maintained in the presence of the consortia (*C. carboxidivorans*/*C. acetobutylicum*), an intense orange colour was obtained in pure cultures of C. *acetobutylicum*, revealing changes in the reduction state of the magnetite. Moreover, despite the black colour was maintained in the presence of *C. carboxidivorans*, magnetite lost the magnetic properties, also indicating alterations in its structure. Unfortunately, those changes were not investigated in detail. In FE treatments, a determination of Fe(III) and Fe(II) revealed all added iron was in the reduced form at the end of the fermentation, and possibly a similar effect should have happened with the structural iron in magnetite.

The use of oxidized iron (Fe(III)) as a sink for an excess of reducing equivalents has been previously observed and is discussed in terms of maintaining metabolic homeostasis and fermentative capacity in the long run (Lovley et al. 2004, List et al. 2019). Moreover, Yang and colleagues already hypothesized the possible reduction of Fe(III) moieties of magnetite particles by *Clostridium* sp. (Yang et al. 2015). *C. acetobutylicum* and other solventogenic species such as *C. beijerinckii* and *C. butyricum* have been found to reduce Fe(III) with glucose as an electron donor (Dobbin et al. 1999, Park et al. 2001, List et al. 2019). However, in the present work, magnetite or Fe(III) additions did not strongly alter the *C. acetobutylicum* performance in pure cultures (butanol and butyrate increases were only significant in MAG treatments), hence suggesting reducing equivalents were similarly distributed into products in all treatments. Contrarily, the carbon fixed into products increased significantly when incubated with magnetite or iron in pure cultures of *C. carboxidivorans* (80% and 74% of added carbon, respectively, compared to 46% of added C in control) (Table 1). More likely, the increase was due to a shift to autotrophy, since pressure decreased drastically in these treatments and CO_2_ + H_2_ was partially consumed.

*Clostridium carboxidivorans* directed carbon assimilation to the production of acetate, butyrate and caproate in the presence of magnetite (Fig. 2), suggesting magnetite could serve as a sink for the excess NADH+H^+^ produced during growth on sugars (<24 h). This effect limited the available reducing equivalents preventing alcohol formation. Similar results to those obtained here were observed in the work of List and coworkers with *C. acetobutylicum* and Fe(III)-citrate, where they found that Fe(III)-citrate could serve as an alternative electron acceptor during glucose fermentation, shifting the electron and carbon flow to the production of butyrate instead of butanol (List et al. 2019). Similarly, the addition of magnetite improved mixotrophy and acetate and butyrate biosynthesis of the acidogenic *C. butyricum*, most probably affecting the activities of key enzymes in the Wood-Ljungdahl pathway (Li et al. 2024a). Plus, higher acid productions when magnetite or other iron oxides were supplemented in anaerobic digesters were also observed and attributed to Fe(III) acting as an electron acceptor for the electron generated during substrate degradation (Su et al. 2020, Wang et al. 2022).

**Figure 4.**
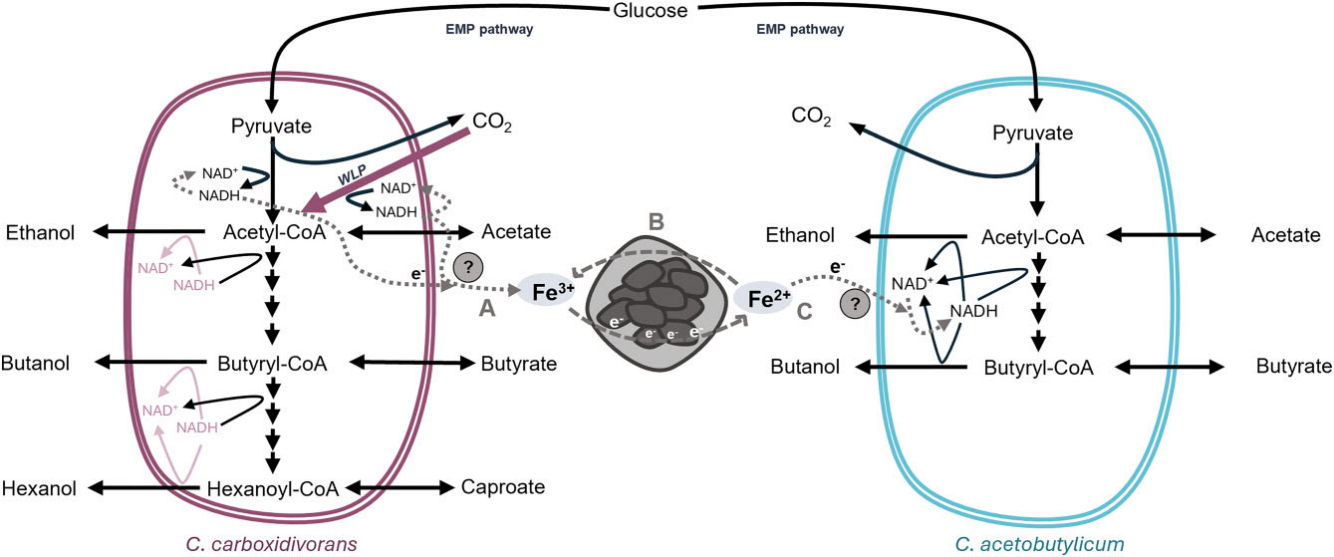
Scheme of the fermentation pathways in *C. carboxidivorans* (left) and *C. acetobutylicum* (right). The hypothesized influence of magnetite supplementation in the electron flow between species is represented in gray. In the absence of magnetite, both species ferment glucose to produce acids and alcohols (black arrows). When magnetite is present, the reduced NADH from *C. carboxidivorans* (produced either during glycolysis or the WoodLjungdahl pathway) can transfer electrons to a soluble (likely riboflavins) electron carrier (pathway A), which in turn reduces the Fe(III) found in the iron complexes in magnetite (pathway B). Later, electrons contained in Fe(II) can be transferred to *C. acetobutylicum* (pathway C, likely through riboflavins) to reduce NAD^+^ to NADH and produce more alcohols.

Magnetite amended co-culture fermentation not only enhanced acid production but also increased the proportion of reducing equivalents incorporated into soluble products. The observed effect is a two-step process. Magnetite is partially used as a sink for reducing equivalents produced by *C. carboxidivorans* during growth on sugar and acid concentration increases (also stimulated by the autotrophic growth), whereas *C. acetobutylicum* will later be able to recover those reducing equivalents and promote the reduction of acids into the corresponding alcohols, mostly occurring at the stationary phase of the fermentation (22 to 44 h) (Fig. 4). Thus, magnetite (or soluble iron at a lower extent) could serve as electric capacitors thus acting as electron acceptors or donors in tested consortia. A similar behaviour has been described with a co-culture of *Rhodopseudomonas palustris* and *Geobacter sulfurreducens*, in which *R. palustris* oxidized magnetite which was then reverted by *G. sulfurreducens* (Byrne et al. 2015). The use of magnetite particles, or similar minerals with equivalent properties, could serve as a mean to increase production yields in co-cultures and opens up the possibility to explore plausible mechanisms at the molecular level in which multi-heme cytochromes may not be involved.

## Conclusions

This study analysed the impact of supplementing three electron-conductive compounds (magnetite, activated carbon, and iron salts) on acid and alcohol production in *Clostridium acetobutylicum* and *C. carboxidivorans* co-culture, as well as their effects when added to each species in pure culture. Results demonstrated that supplementing with magnetite and iron salts improved carbon fixation by enhancing the autotrophy of *C. carboxidivorans*. However, the addition of MAG and FE in *C. carboxidivorans* pure cultures only enhanced acid production, while it decreased ethanol concentrations. Contrarily, in the co-culture, magnetite acted as both an electron sink and source, leading to increased concentrations of acids and alcohols. More precisely, magnetite boosted the production of longer-chain acids and alcohols (C4 and C6), which reached their highest production levels after 44 h of fermentation. Our findings suggest that iron availability, rather than conductivity alone, may be the primary factor influencing fermentation outcomes under magnetite supplementation. These results provide valuable insights into the role of conductive materials composition in promoting redox reactions and electron transfer.

## Supplementary Material

fiaf090_Supplemental_File
