# Supplementary material for: Electron conductive compounds alter fermentative pathways and cooperation in Clostridium carboxidivorans and Clostridium acetobutylicum in co-culture
Source: FEMS Microbiol Ecol. 2025 Sep 16;101(10):fiaf090. doi: 10.1093/femsec/fiaf090 (PMC12451442; doi:10.1093/femsec/fiaf090)
Supplement: fiaf090_Supplemental_File [file fiaf090_supplemental_file.docx]

**Electron conductive compounds supplementation alters fermentative pathways and cooperation in *Clostridium carboxidivorans* and *Clostridium acetobutylicum* in co-culture**

Laura Feliu-Paradeda ^a^, Sebastià Puig ^b^ and Lluís Bañeras ^a^

^a^ Molecular Microbial Ecology Group, Institute of Aquatic Ecology, University of Girona, Carrer Maria Aurèlia Capmany 40, E-17003 Girona, Spain.

^b^ LEQUiA, Institute of the Environment, University of Girona, Carrer Maria Aurèlia Capmany 69, E-17003 Girona, Spain

*Corresponding author: [lluis.banyeras@udg.edu](mailto:lluis.banyeras@udg.edu)

**Supplementary Figures**

**
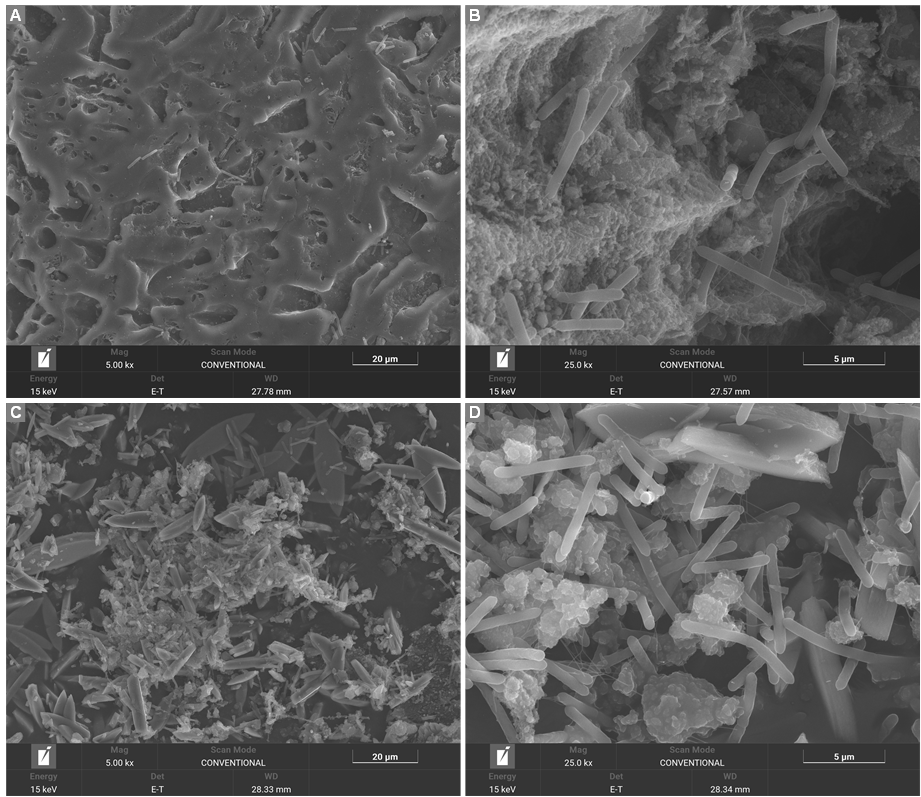
**

**Supplementary Figure S1.** SEM images of *Clostridium acetobutylicum/Clostridium carboxidivorans* co-culture cells with activated carbon (A, B) and magnetite (C, D) at the end of the fermentation experiments. B and D are magnified images of A and C, respectively.


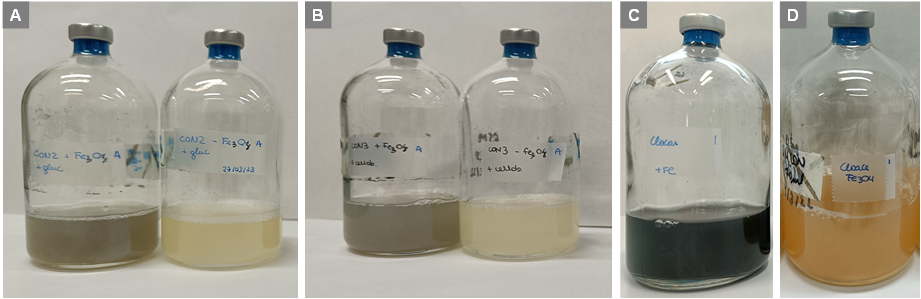


**Supplementary Figure S2.** Coloration in magnetite supplemented bottles after 44 hours of fermentation in *C. acetobutylicum/C. carboxidivorans* co-culture (A), the triplet consortium (B), *C. carboxidivorans* (C) and *C. acetobutylicum* (D). Consortia bottles containing magnetite are placed on the left, whereas control bottles are on the right.
